# Surgical Apgar score is strongly associated with postoperative ICU admission

**DOI:** 10.1038/s41598-020-80393-z

**Published:** 2021-01-08

**Authors:** Ying-Chun Lin, Yi-Chun Chen, Chen-Hsien Yang, Nuan-Yen Su

**Affiliations:** 1 Department of Anesthesiology, Mackay Memorial Hospital, Taipei, 104 Taiwan; 2 Mackay Junior College of Medicine, Nursing and Management, Taipei, 112 Taiwan; 3 Mackay Medical College, Taipei, 252 Taiwan; 4 College of Medicine, Graduate Institute of Clinical Medicine, National Taiwan University, Taipei, 100 Taiwan

**Keywords:** Health care, Medical research

## Abstract

Immediate postoperative intensive care unit (ICU) admission can increase the survival rate in patients undergoing high-risk surgeries. Nevertheless, less than 15% of such patients are immediately admitted to the ICU due to no reliable criteria for admission. The surgical Apgar score (SAS) (0–10) can be used to predict postoperative complications, mortality rates, and ICU admission after high-risk intra-abdominal surgery. Our study was performed to determine the relationship between the SAS and postoperative ICU transfer after all surgeries. All patients undergoing operative anesthesia were retrospectively enrolled. Among 13,139 patients, 68.4% and < 9% of whom had a SASs of 7–10 and 0–4. Patients transferred to the ICU immediately after surgery was 7.8%. Age, sex, American Society of Anesthesiologists (ASA) class, emergency surgery, and the SAS were associated with ICU admission. The odds ratios for ICU admission in patients with SASs of 0–2, 3–4, and 5–6 were 5.2, 2.26, and 1.73, respectively (P < 0.001). In general, a higher ASA classification and a lower SAS were associated with higher rates of postoperative ICU admission after all surgeries. Although the SAS is calculated intraoperatively, it is a powerful tool for clinical decision-making regarding the immediate postoperative ICU transfer.

## Introduction

In Taiwan, more than 14.9 million patients underwent surgery in 2015, including 4.6 million who underwent in-hospital surgeries^1^. Previous studies have shown that patients undergoing high-risk surgeries account for more than 80% of postoperative deaths; nevertheless, less than 15% were admitted to the intensive care unit (ICU) immediately after the operation^2^. Patients who received intensive care immediately following the operation had a lower level of morbidity^3^.

Unplanned postoperative ICU admission (i.e., ICU admission is not part of the preoperative plan) was associated with a significantly higher rate of mortality^3,4^; however, ICU admission without any clear indications may increase the postoperative hospital length of stay and associated costs^5^. Thus, adopting adequate criteria for ICU admission is crucial. Previous studies have reported indicators based on retrospective analyses of pediatric patients or specific surgeries, such as major pulmonary resection, urologic surgery, joint replacement, emergency operation, and degenerative spinal surgery^6–11^, but none of these indicators can be generally applied.

The surgical Apgar score (SAS), which has been widely discussed over the past 10 years, is a simple formula that uses intraoperative hemodynamics and blood loss to predict the postoperative complications and mortality rate (Table 1)^6^. The SAS was positively correlated with postoperative complications and/or mortality^7^ in patients undergoing vascular surgery,^8^ noncardiac surgery^9^, general surgery^8^, esophagectomy^10^, colorectal resection^11^, emergency abdominal surgery^12^, elective laparoscopic cholecystectomy^13^, hepatectomy for hepatocellular carcinoma^14^, liver transplantation^15^, general oncologic surgery^8,16,17^, laparotomy gynecological surgery^18^, radical or partial nephrectomy for renal mass excision^19^, radical protectomy^20^, hip or knee arthroplasty^21^, transfemoral amputation^22^, lumbar fusion for degenerative spine diseases^23^, intracranial meningioma^24^, and head and neck cancer^13,25^.

**Table 1:** Calculation of the surgical Apgar score, ranging from 0 to 10 points, with lower scores associated with worse postoperative outcomes.

	Surgical Apgar Score, number of points				
	0	1	2	3	4
Estimated blood loss (ml)	> 1000	601–1000	101–600	≤ 100	–
Lowest MAP (mmHg)	< 40	40–54	55–69	≥ 70	–
Lowest HR (beats/min)	> 85	76–85	66–75	56–65	≤ 55

*MAP* mean arterial pressure, *HR* heart rate.

Due to its ability to predict postoperative complications and mortality, the SAS is considered a potential tool for the prediction of postoperative ICU admission. Previous studies have concluded that the SAS is correlated with ICU admission after high-risk intra-abdominal surgery^26^ and general surgery^27^. However, its ability to predict ICU admission after other operations remains unknown. This study was performed to determine the relationship between the SAS and postoperative ICU transfer after all surgeries.

## Results

### Patient characteristics

Between January and June 2017, a total of 13,297 adult patients were enrolled in our cohort, and after the exclusion of patients with incomplete data (n = 142) and repeated surgeries (n = 16), 13,139 patients were included in the final analysis. The cohort was mostly composed of patients aged ≤ 50 years (51.6%), with a mean age of 47.9 years (± 20.6). Among all patients, 58.5% were female, 91.3% underwent elective surgery, and most were categorized into American Society of Anesthesiologists (ASA) class II (61.2%). Approximately 27% of the patients underwent gynecological surgery, and 15%, 13%, and 12% underwent orthopedic, general, and urologic surgery, respectively (Table 2). Approximately half of the cohort had an SAS of 7–8, and < 9% had an SAS of 0–4 (Table 2).

**Table 2:** Patient characteristics and frequency of admission to the ICU.

Patient characteristics	Total number of patients, n (% of all patients)	Immediate ICU admission, n (% of total in each stratum)	*P*-value
Total number of patients, n	13,139	1020 (7.8)	
**Age (years)**			< 0.001
< 50	6,783 (51.6)	275 (4.1)	
50–59	2,255 (17.2)	183 (8.1)	
60–69	2,190 (16.7)	284 (13.0)	
70–79	1255 (9.6)	156 (12.4)	
≥ 80	656 (5.0)	122 (18.6)	
**Gender**			< 0.001
Female	7688 (58.5)	402 (5.2)	
Male	5451 (41.5)	618 (11.3)	
**ASA**			< 0.001
I	2160 (16.4)	25 (1.2)	
II	8041 (61.2)	241 (3.0)	
III	2836 (21.6)	669 (23.6)	
IV–V	102 (0.8)	85 (83.3)	
**Procedure**			< 0.001
Emergency	1144 (8.7)	229 (20.0)	
Elective	11,995 (91.3)	791 (6.6)	
**Type of surgery**			< 0.001
GS	1681 (12.8)	141 (8.4)	
Gyn	3524 (26.8)	45 (1.3)	
Obs	18 (0.1)	0 (0.0)	
Ped	656 (5.0)	33 (5.0)	
Ortho	2004 (15.3)	28 (1.4)	
NS	584 (4.4)	145 (24.8)	
Uro	1567 (11.9)	60 (3.8)	
PS	530 (4.0)	65 (12.3)	
CRS	800 (6.1)	117 (14.6)	
CVS	124 (0.9)	73 (58.9)	
CS	480 (3.7)	233 (48.5)	
Oph	117 (0.9)	0 (0.0)	
ENT	743 (5.7)	41 (5.5)	
Dent	17 (0.1)	0 (0.0)	
OS	290 (2.2)	37 (12.8)	
Pain	2 (0.0)	0 (0.0)	
Other	2 (0.0)	2 (100.0)	
**SAS**			< 0.001
0–2	52 (0.4)	24 (46.2)	
3–4	1112 (8.5)	189 (17.0)	
5–6	2993 (22.8)	373 (12.5)	
7–8	6452 (49.1)	354 (5.5)	
9–10	2530 (19.3)	80 (3.2)	

*ICU* intensive care unit, *ASA* American Society of Anesthesiologists, *SAS* surgical Apgar score, *GS* general surgery, *Gyn* gynecology, *Obs* obstetrics, *Ped* pediatrics, *Ortho* orthopedics, *NS* neurosurgery, *Uro* urology, *PS* plastic surgery, *CRS* colorectal surgery, *CVS* cardiovascular surgery, *CS* chest surgery, *Oph* ophthalmology, *ENT* otorhinolaryngology, *Dent* dentistry, *OS* oral surgery.

### Frequency of admission to ICU

In this cohort, 1020 patients (7.8%) were transferred directly from the operating room or post-anesthesia care unit (PACU) to the ICU after the operation. There were statistically significant differences in the patient characteristics between the groups of patients who were and were not immediately admitted to the ICU (Table 2). Factors associated with a higher frequency of ICU admission included older age, male sex, emergency surgery, and higher ASA class. The type of operation also affected the rates of ICU admission, and more than half of the patients who underwent cardiovascular surgery and almost half of those who underwent thoracic surgery were admitted to the ICU immediately, whereas none of the patients who underwent obstetric, ophthalmic, dental, or pain-related procedures were admitted to the ICU (Table 1). As the SAS decreased from 9–10 to 0–1, the frequency of ICU admission progressively increased from 3.2 to 46.2% (P < 0.001, Table 2).

### Variables associated with immediate ICU admission

According to the multivariate logistic regression model, age, sex, ASA classification, emergency surgery, and SAS were associated with the ICU admission rate (Table 3). Compared to patients classified as ASA I, those classified as ASA III and ASA IV had higher odds of ICU admission (adjusted OR, 13.97; 95% confidence interval [CI] 9.88–19.76; P < 0.01 and limitless, respectively). Males were more likely than females to be admitted to the ICU after surgery (adjusted OR, 1.56; 95% CI 1.36–1.8). In addition, patients receiving emergency operations were more likely to be postoperatively admitted to the ICU than those who underwent elective operations (adjusted OR, 1.83; 95% CI 1.5–2.24; P < 0.001). After adjusting for age, sex, ASA class, and the emergency nature of the surgery, a relatively lower SAS was still strongly associated with ICU admission when compared to the reference group of patients with a SAS of 7 to 8 (adjusted OR, 5.21; 95% CI 2.49–10.88; P < 0.001).

**Table 3:** Variables associated with immediate ICU admission as determined by univariate and multivariate analyses, with unadjusted and adjusted odds ratios (ORs) and 95% confidence intervals (CIs).

Patient characteristics	Unadjusted		Adjusted	
	OR (95% CI)	*P*-value	OR (95% CI)	*P*-value
**Age (years)**				
< 50	Reference		Reference	
50–59	2.09 (1.72–2.54)	< 0.01	1.80 (1.45–2.24)	< 0.001
60–69	3.53 (2.96–4.19)	< 0.01	2.10 (1.72–2.57)	< 0.001
70–79	3.36 (2.73–4.13)	< 0.01	1.31 (1.03–1.66)	0.03
≥ 80	5.41 (4.29–6.81)	< 0.01	1.48 (1.14–1.93)	< 0.001
**Gender**				
Female	Reference		Reference	
Male	2.32 (2.03–2.64)	< 0.01	1.56 (1.36–1.80)	< 0.001
**ASA**				
I	Reference		Reference	
II	2.38 (1.75–3.52)	< 0.01	1.86 (1.32–2.63)	< 0.001
III	24.8 (17.69–34.75)	< 0.01	13.97 (9.88–19.76)	< 0.001
IV–V	∞	–	∞	–
**Procedure**				
Emergency	3.54 (3.02–4.17)	< 0.01	1.83 (1.50–2.24)	< 0.001
Elective	Reference		Reference	
**SAS**				
0–2	5.7 × 10^181^ (1.5 × 10^178^–2.1 × 10^185^)	< 0.01	5.21 (2.49–10.88)	< 0.001
3–4	3.53 (2.93–4.26)	< 0.01	2.26 (1.81–2.83)	< 0.001
5–6	2.45 (2.11–2.86)	< 0.01	1.73 (1.47–2.04)	< 0.001
7–8	Reference	< 0.01	Reference	< 0.001
9–10	0.56 (0.44–0.72)	< 0.01	0.50 (0.40–0.63)	< 0.001

*ICU* intensive care unit, *ASA* American Society of Anesthesiologists, *SAS* surgical Apgar score.

∞: infinity.

^†^–: unable to calculate.

## Discussion

The SAS is a simple formula that uses intraoperative hemodynamics and blood loss to predict postoperative complications and mortality rates^6^. The SAS was found to be positively correlated with postoperative complications and/or mortality in patients undergoing a wide variety of procedures^7–25^. Due to its ability to predict postoperative complications and mortality, the SAS might also be useful for the prediction of postoperative ICU admission. Our study demonstrated that the SAS was strongly associated with postoperative ICU transfer after all surgeries.

We found that a low SAS was strongly associated with a higher frequency of immediate postoperative ICU transfer after all surgeries. In general, our results were similar to those of previous studies; for example, associations among postoperative ICU admission, the SAS and specific patient characteristics have been shown in other studies^26,27^; however, there were several fundamental differences among the studies that may explain the minor differences in the results. Previous studies focused on high-risk intra-abdominal surgery and general surgical procedures, excluding trauma-related and laparoscopic surgeries, while our study enrolled all patients regardless of the surgery type. Furthermore, the study by Glass et al. was carried out at a veterans’ health center and had a much higher proportion of male patients (89.6%) than those in the study by Sobol et al. and our study (51.4% and 41.5%, respectively). This study was carried out at a single medical center that is well known for obstetrics and gynecology; thus, we had a higher proportion of female patients and a younger population (47.9 ± 20.6 years) than those in the study by Glass et al. (61.8 ± 14.5 years) and Sobol et al. (59 ± 15.6 years). With regard to the baseline characteristics, the study by Glass et al. classified most patients in ASA III, despite admission to the ICU, while most of our patients and those in the study by Sobol et al. were categorized as ASA II. Both the present study and that by Sobol et al. reported that most patients admitted postoperatively to the ICU were classified as ASA IV or V. Although Glass et al. excluded trauma cases, the authors still reported the highest rate of emergency operations. All studies were retrospective in design; however, our study included 1.5 times more patients (n = 13,139) than the other studies (n = 1517 and 8501)^26,27^.

Overall, most patients had SASs of 7–8 (49.1%) and 9–10 (19.3%); however, those with SASs of 0–2 (46.2%) and 3–4 (17.0%) accounted for the majority of the patients admitted postoperatively to the ICU. This result was similar to that in the study by Sobol et al., which reported prevalences of SASs of 7–8 and 9–10 of 49.3% and 23.4%, respectively, and prevalences of ICU admission of 56.5% and 32.2% in patients with SASs of 0–2 and 3–4, respectively. However, Glass et al. reported a similar distribution of mostly SASs of 7–8 and 9–10 in patients sent to a general ward or the ICU. The difference may be due to older patient age and more severe comorbidities specific to the selected patients; however, this suggestion warrants further investigation.

Similar to previous studies^26,27^, we found higher adjusted ORs for ICU admission associated with older age, higher ASA class, emergency operation, and low SAS. Although the trends in the overall results were similar, the specific values were not. For example, Sobol et al. reported an OR of 14.4 for ICU admission in patients with SAS of 0–2, while our study reported an OR of 5. The inconsistency among these values may indicate differences among the patient characteristics and their baseline risk. Previous studies also noted an increased OR for ICU admission in patients with moderate to severe anemia, receiving anesthesia for more than 6 h or undergoing specific procedures, for example, esophagectomy, hepatobiliary and pancreatic surgery, cystectomy, prostatectomy, major vascular surgery, and exploratory laparotomy^26,28^. For those with a history of chronic obstructive pulmonary disease (COPD), an increased OR for ICU transfer has also been reported^27^. In retrospective analysis, patients with congestive heart failure, chronic kidney disease, peripheral vascular diseases, vascular diseases or higher BMI (i.e. BMI > 30) are found to be associated with unexpected postoperative ICU admission^29,30^. Some procedure-specific risk factors are also linked to unplanned ICU admission, for example, revision surgery for total hip arthroplasty or total knee arthroplasty^31^. In brief, most studies are retrospective in design and show that age, BMI, ASA classification, type of procedure (or surgical risk), duration of surgery, emergency surgery, revision operation, estimated blood loss, anemic status and specific comorbidities may relate to postoperative ICU admission. Whether specific factors or composite scoring systems predict postoperative ICU admission best is still under passionate discussion.

In this study, we mostly included Asian patients, while other studies included higher proportions of Caucasian and African American individuals^26,27^. Although the studies were carried out in different countries, with different systems and with different ethnicities that could have influenced the results, they all showed a similar trend in ICU admission. In 2010, Haynes et al. enrolled patients from eight different countries and concluded that the SAS was valid across different ethnicities^32^. Thus, we believe that the association between ICU admission and a low SAS will be confirmed in future studies, regardless of the ethnicity of the study population.

Most studies, including ours, aimed to validate the SAS as a predictor of postoperative ICU admission^26,27^. However, a prospective study using the SAS as a clinical aid yielding similar positive results is needed before the SAS can be utilized in daily practice with confidence. One randomized controlled trial examined the effect of the use of the SAS as a clinical tool for determining ICU transfer^33^. Although the study only provided clinicians with the score before any clinical decisions were made rather than instructing them to use the SAS as a guide, this trial provided a high level of evidence of the clinical utility of the SAS and was designed to reflect real-world clinical practice, preserving physicians’ freedom with regard to final decision-making. When the SAS was used, the rate of postoperative ICU admission was slightly, but insignificantly, increased, and the occurrences of morbidity and mortality remained unchanged^33^. The similar rates of morbidity and mortality suggest that appropriate ICU admission immediately after surgery might reduce morbidity and mortality^3,4^. Nevertheless, the study did not address whether the use of the SAS can decrease unplanned and delayed ICU admission, which are associated with higher mortality^3,4^.

There were several limitations of our study. First, the patients were collected from a single medical center in Taiwan, and more than a quarter of the patients underwent obstetrics and gynecology surgery; therefore, the generalizability of our results to other systems and/or populations is unknown. Second, this study was retrospective in nature. Our findings showed an association between current decision-making regarding ICU admission and the SAS. Other influential factors, such as patient comorbidities, family expectations, surgeon preferences, and issues pertaining to the health care system, were not evaluated. Whether the SAS can serve as a proxy for a combination of these considerations is not known. Third, we did not analyze delayed ICU admission; thus, it is unknown whether the use of the SAS can prevent unexpected ICU admission by facilitating the immediate provision of intensive care following the operation. Fourth, our study did not collect the morbidity and mortality rates. Thus, it is unknown whether ICU admission can truly improve clinically significant outcomes, such as morbidity and mortality, as shown in previous studies. Finally, an accurate SAS value can only be calculated at the end of surgery; however, earlier triage is important for ICU resource allocation. If the ICU bed is not reserved in advance, and ICU admission is not possible due to limited resources, patient outcomes could be significantly impacted.

In conclusion, our study showed that intraoperative hemodynamics and blood loss may affect postoperative transfer to the ICU and that the SAS is strongly associated with postoperative ICU admission in patients undergoing all types of surgeries. However, as the SAS cannot be calculated in advance, the use of the SAS might postpone the timing of decision-making and impact patient outcomes. Despite this, the SAS remains a powerful tool that can be used to facilitate clinical decision making with regard to the immediate transfer of patients to the ICU after surgery.

## Methods

### Patient selection

All adult patients undergoing anesthesia during operations at Mackay Memorial Hospital (MMH, Taipei, Taiwan) were retrospectively enrolled from January to June 2017. This study was carried out in accordance with the regulations of the MMH Institutional Review Board (IRB), which approved the study protocol and waived the need to obtain written consent from the patients (19MMHIS118e). Patient characteristics, including age, sex, regular or emergency surgery, type of operation, and ASA class, were obtained from the anesthesia information management system. For patients receiving repeated or sequential surgeries during a single hospital admission, only the first operation was included. Immediate ICU admission was extracted from the MMH electronic clinical information system.

Immediate ICU admission was defined as transfer directly from the operating room or the PACU to the ICU. At our institution, all patients were sent to the PACU after the operation unless they required a ventilator or advanced life support, such as an intra-aortic balloon pump (IABP) or extracorporeal membrane oxygenation (ECMO). The patients were then sent to the ICU or the ward as planned. Our ICU provides a 1:2 nurse-to-patient ratio and offers advanced monitoring (i.e., arterial blood pressure and pulse-induced contour cardiac output) and life support (i.e., mechanical ventilation, renal replacement therapy, administration of vasopressors and inotropic drugs, IABP, and ECMO). Decisions about patient destination after the operation were made by both the anesthesiologist and surgeon.

### Data collection

The following intraoperative data were extracted from the anesthesia information management system and used to calculate the SAS: maximum estimated blood loss (EBL), lowest mean arterial pressure (MAP), and lowest heart rate (HR) (Table 1). The raw data were acquired as an Excel file, from which were retrieved the relevant data. The SAS was calculated based on the absolute values of the EBL, MAP, and HR.

### Statistical analysis

The patient characteristics and outcomes for the entire cohort were summarized first. The variables were categorized to improve the discriminatory power. For example, age was grouped into the categories, namely, < 50, 50–59, 60–69, 70–79, and ≥ 80 years; ASA classes IV and V were combined into one class; and the total SAS was divided into five groups, namely, from 0 to 2, 3–4, 5–6, 7–8, and 9–10. The median SAS group from 7 to 8 was selected as the reference group, as in previous studies^26,28^. For categorical variables, the chi-square test was used to assess differences between groups.

To evaluate potential associations between each variable and postoperative ICU admission, we used univariate logistic regression models with age, sex, ASA physical status, emergency surgery, operative department, and SAS. A multivariate logistic regression model was created to evaluate the adjusted odds ratios (ORs).

Database management and statistical analyses were carried out with SPSS 25.0 (IBM Corp. Armonk, NY, USA). A P-value < 0.05 was considered statistically significant.
